# Supplementary material for: TrkB-Induced Inhibition of R-SMAD/SMAD4 Activation is Essential for TGF-β-Mediated Tumor Suppressor Activity
Source: Cancers (Basel). 2020 Apr 23;12(4):1048. doi: 10.3390/cancers12041048 (PMC7226331; doi:10.3390/cancers12041048)
Supplement: Supplementary file 1 [file cancers-12-01048-s001.pdf]

Supplementary Figures

TrkB-Induced Inhibition of R-SMAD/SMAD4 Activation is Essential for TGF-β-Mediated Tumor Suppressor Activity

Min Soo Kim and Wook Jin

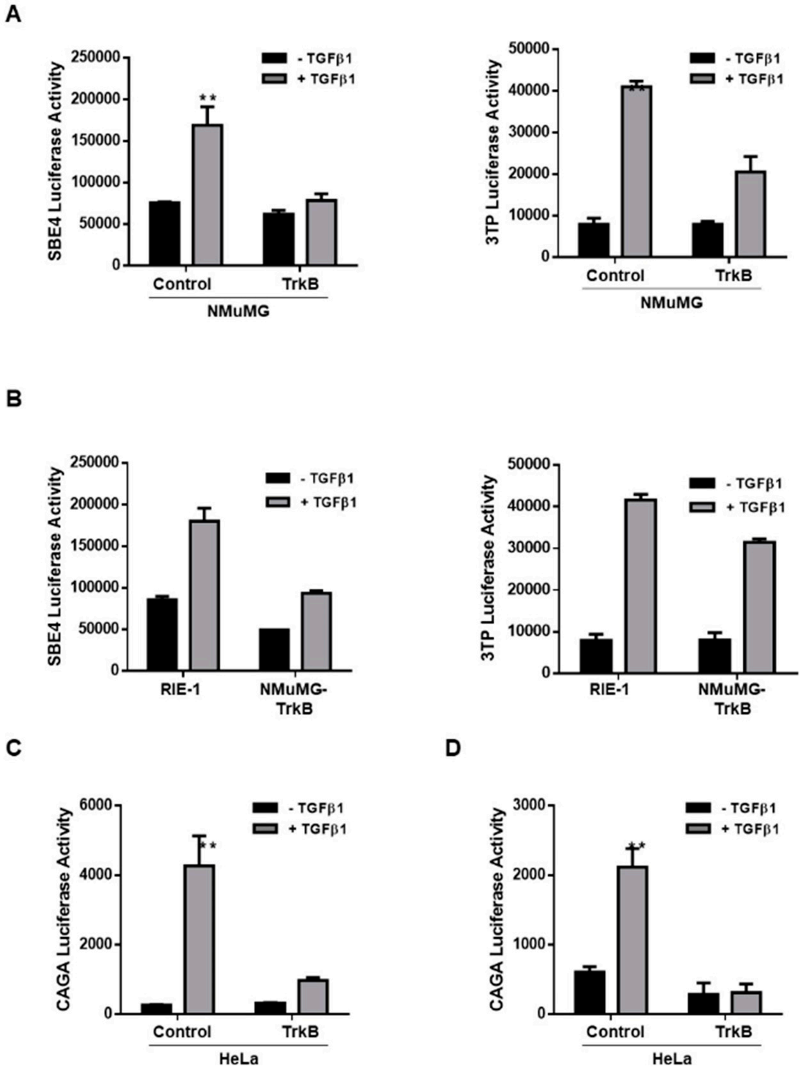

Figure S1. TrkB overexpression inhibits TGF-β signaling. (**A**) Luciferase reporter assay of TGF-β1-responsive SBE4 and 3TP in NMuMG cells transfected with the TrkB. ** Control versus treatment with TGF-β1, $p < 0.05$. $n = 3$. (**B**) Luciferase reporter assay of TGF-β1-responsive SBE4 and 3TP in NMuMG or NMuMG-TrkB cells. **Control versus treatment with TGF-β1, $p < 0.05$. $n = 3$. (**C**) Luciferase reporter assay of SMAD3-dependent $(CAGA)_{12}$-Luc in HeLa cells transiently transfected with the TrkB. **Control versus treatment with TGF-β1, $p < 0.05$. $n = 3$. (**D**) Luciferase reporter assay of SMAD3-dependent $(CAGA)_{12}$-Luc in HeLa or HeLa-TrkB cells. **Control versus treatment with TGF-β1, $p < 0.05$. $n = 3$.

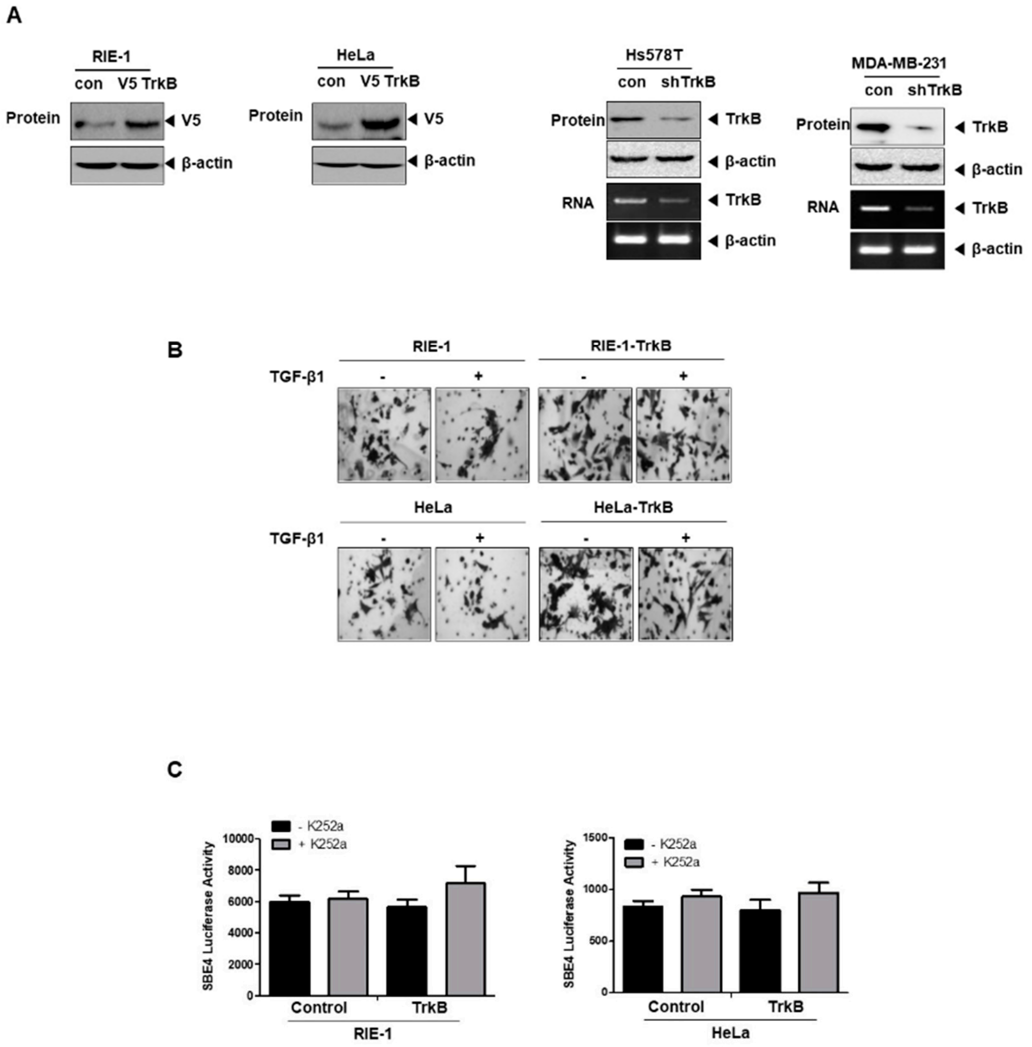

Figure S2. TrkB induces the migaration ability of RIE-1 and HeLa cells**.** (**A**) Western blot analysis of V5-TrkB expression in HeLa, RIE-1, HeLa-TrkB, and RIE-1-TrkB cells, and RT-PCR and western blot analysis of TrkB expression in Hs578T, MDA-MB-231 control-shRNA or TrkB-shRNA cells. β-actin were used as loading controls (**B**) Migration assay of HeLa, RIE-1, HeLa-TrkB, and RIE-1-TrkB cells after treatment of TGF-β1 (5 ng/mL) (**C**) Luciferase reporter assay of K252a-responsive SBE in HeLa, RIE-1, HeLa-TrkB, and RIE-1-TrkB cells.

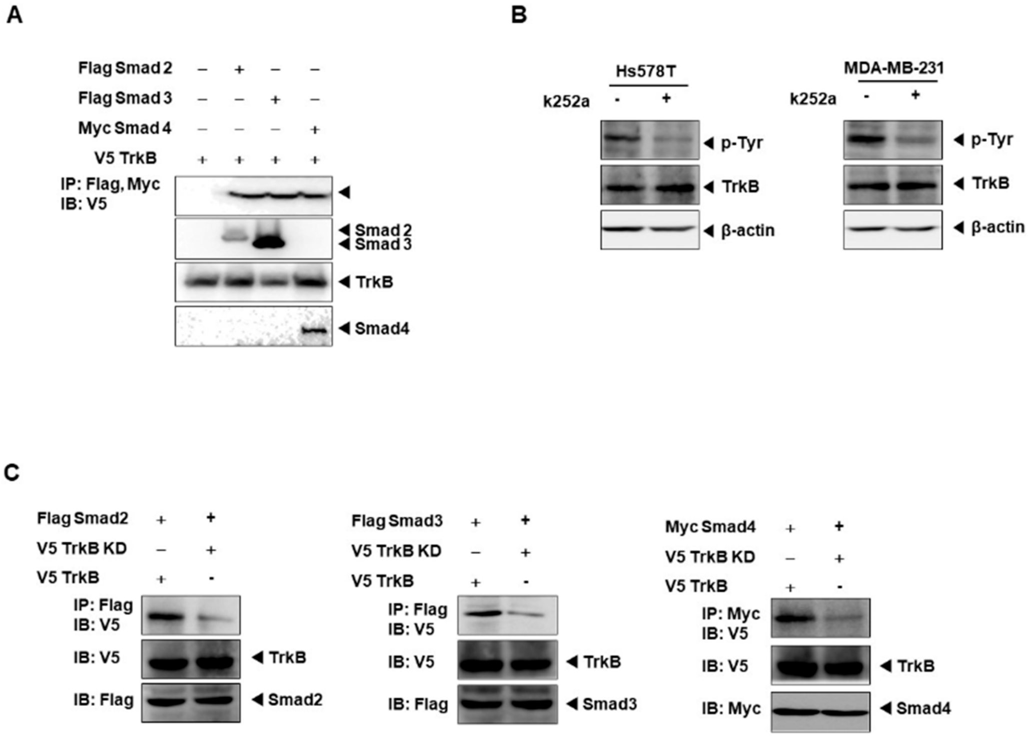

Figure S3. The kinase domain of TrkB essential for the complex TrkB/SMADs formation. (**A**) TrkB directly interacts with SMAD2, SMAD3, and SMAD4. Western blot analyses of V5-TrkB following immunoprecipitation of Flag-SMAD2, Flag-SMAD3, or Myc-SMAD4 from a whole-cell extract of transfected 293T cells. (**B**) Western blot analysis of TrkB activation in Hs578T and MDA-MB-231 cells with or without 100 nM K252a treatment. β-actin was used as a loading control. (**C**) Immunoblot analysis of whole-cell lysates and immunoprecipitates derived from 293T cells transfected with V5-TrkB or V5-TrkB KD constructs and Flag-SMAD2, Flag-SMAD3, or Myc-SMAD4 constructs as indicated.

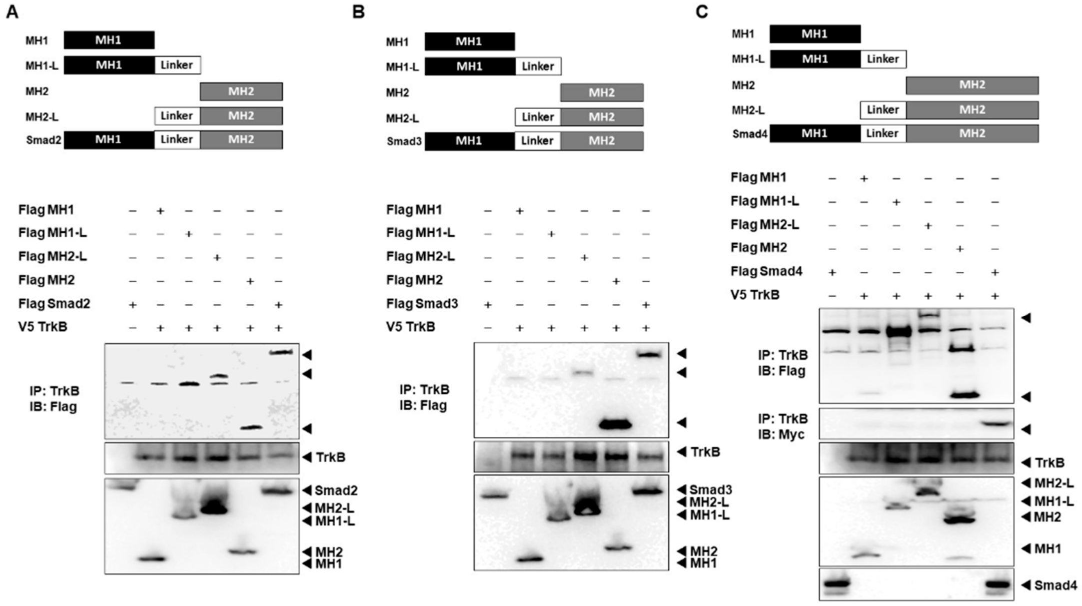

Figure S4. TrkB directly interacts with the MH2 domain of SMADs. (**A**) Western blot analyses of V5-TrkB following immunoprecipitation of Flag-SMAD2, or Flag-SMAD2 deletion constructs from a whole-cell extract of transfected 293T cells. (**B**) Western blot analyses of V5-TrkB following

immunoprecipitation of Flag-SMAD3, or Flag-SMAD3 deletion constructs from a whole-cell extract of transfected 293T cells. (**C**) Western blot analyses of V5-TrkB following immunoprecipitation of Myc-SMAD4, or Flag-SMAD4 deletion constructs from a whole-cell extract of transfected 293T cells.

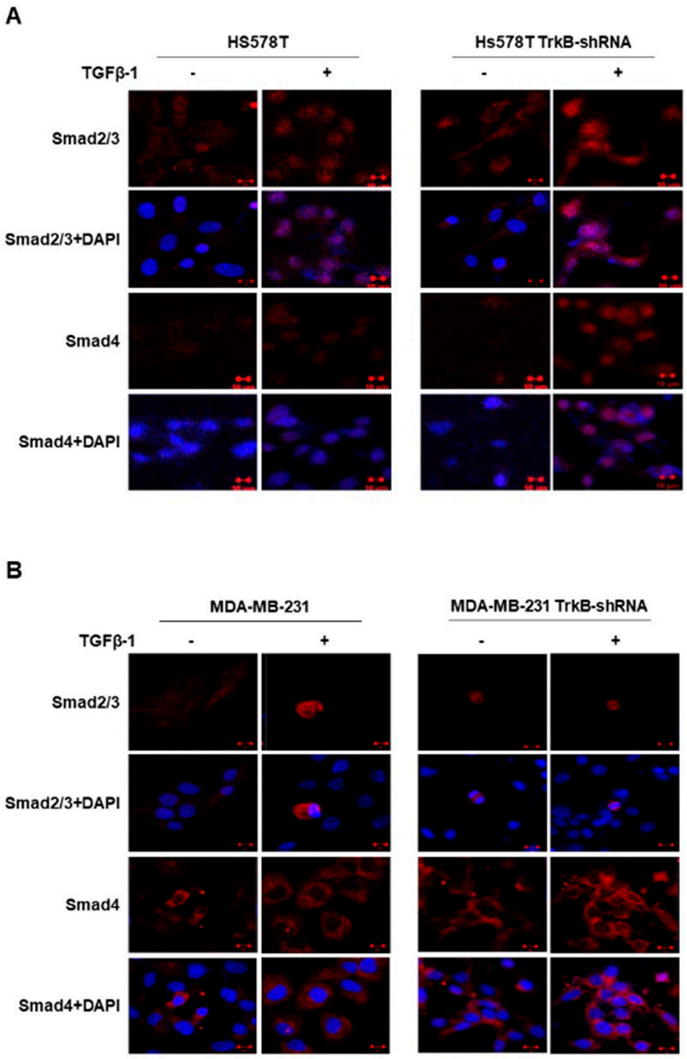

Figure S5. The loss of TrkB enhances nuclear retention of SMAD2, SMAD3, and SMAD4. (**A**) & (**B**) Immunofluorescence staining of SMAD2, SMAD3, and SMAD4 in MDA-MB-231 and Hs578T control-shRNA or TrkB-shRNA cells after treatment with TGF-β1 (5 ng/mL) for 1 hour. The scale bar represents 10 μm.

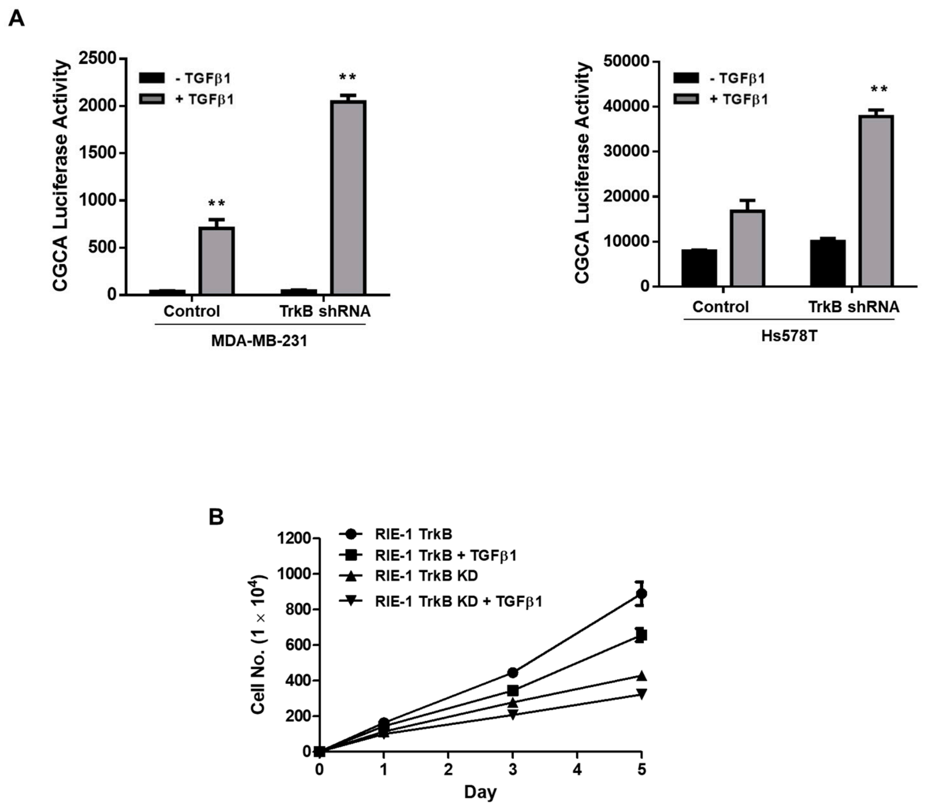

Figure S6. TGF-β-mediated growth inhibition induced by Loss of kinase activity of TrkB. (**A**) Luciferase reporter assay of SMAD3-dependent $(CAGA)_{12}$-Luc in MDA-MB-231- and Hs578T control-shRNA or TrkB-shRNA cells. **Control versus treatment with TGF-β1, $p < 0.05$. $n = 3$. (**B**) cell growth of RIE-1-TrkB, and RIE-1-TrkB KD cells after treatment of TGF-β1 (5 ng/mL). Each data point represents the mean number of cells counted in three dishes.

Detailed Information about Western Blot

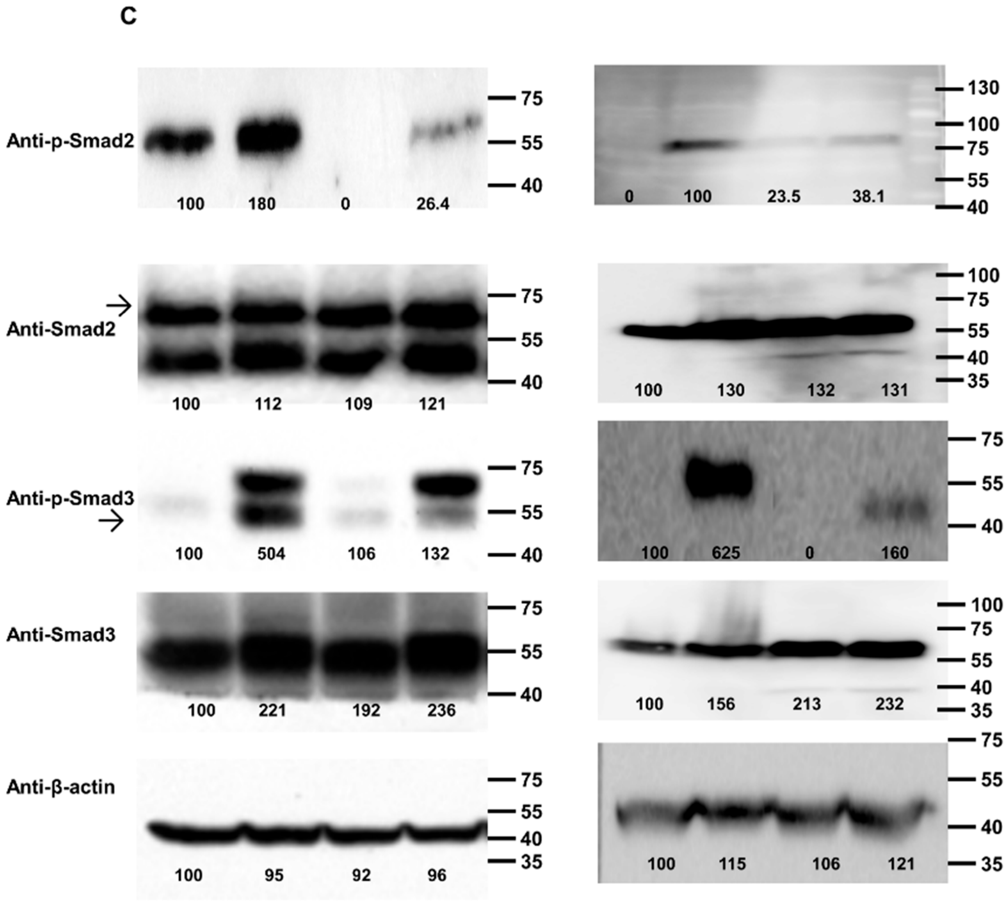

Detailed information about western blot of Figure 1C.

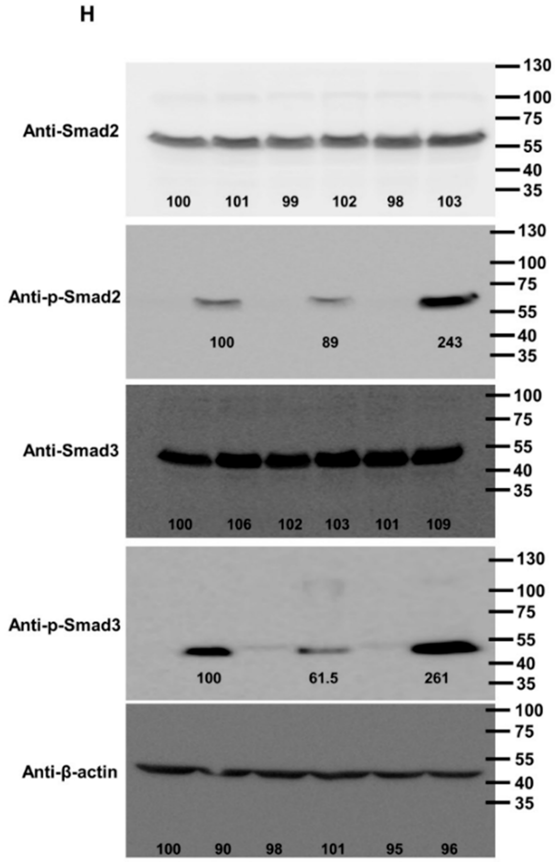

Detailed information about western blot of Figure 2H.

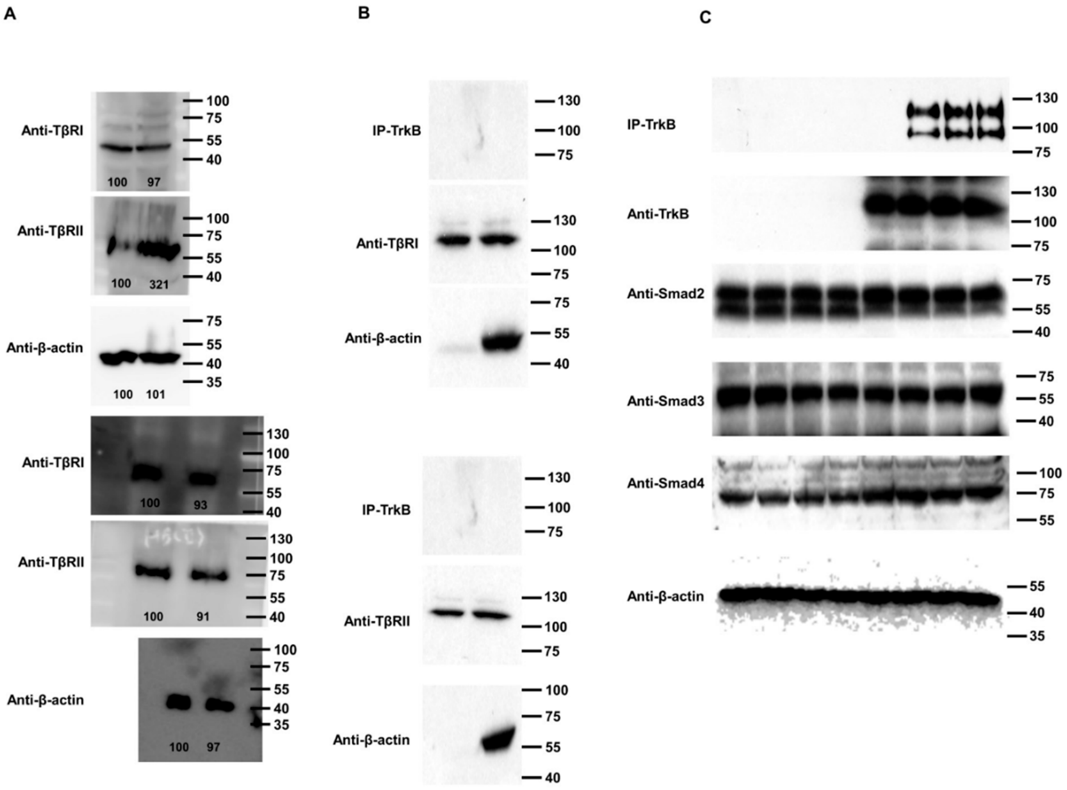

Detailed information about western blot of Figure 3A–C.

D

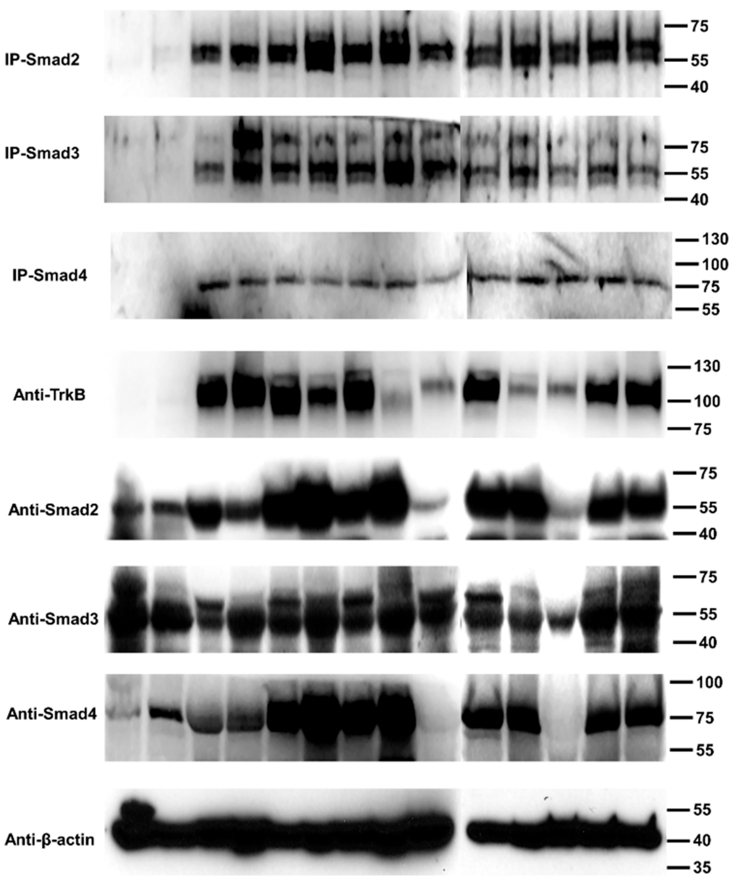

Detailed information about western blot of Figure 3D.

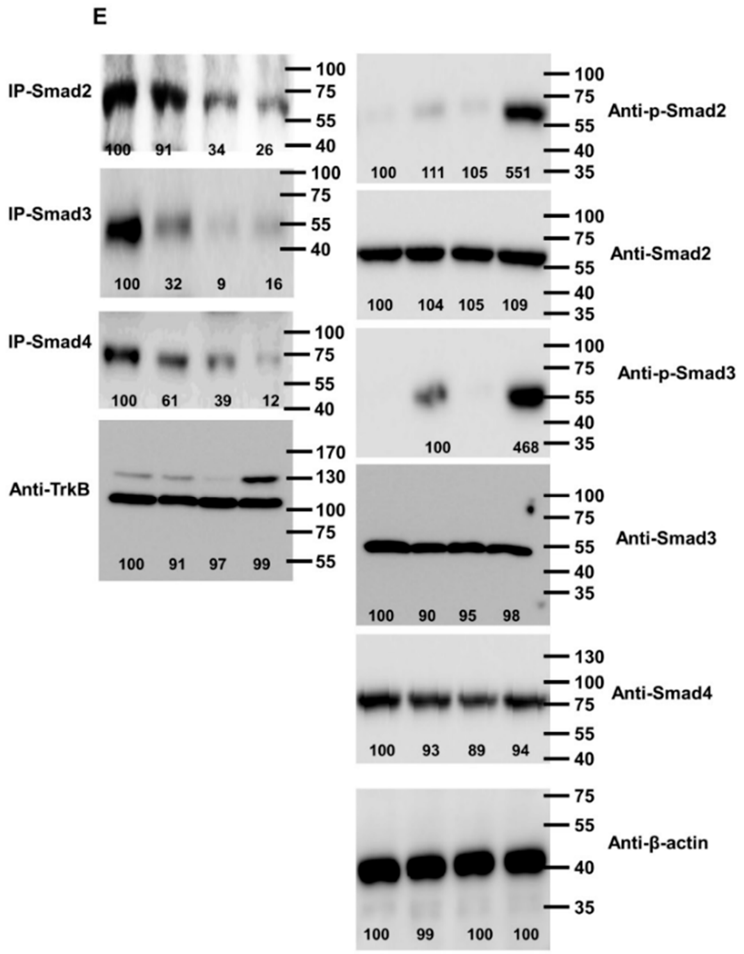

Detailed information about western blot of Figure 3E.

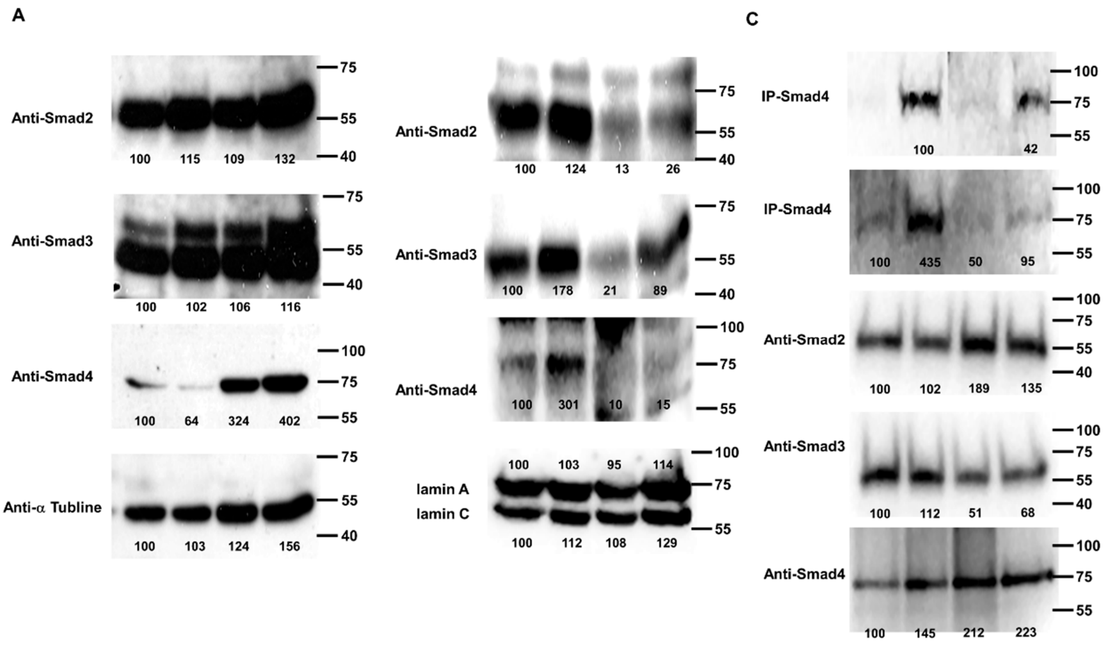

Detailed information about western blot of Figure 4A,C.

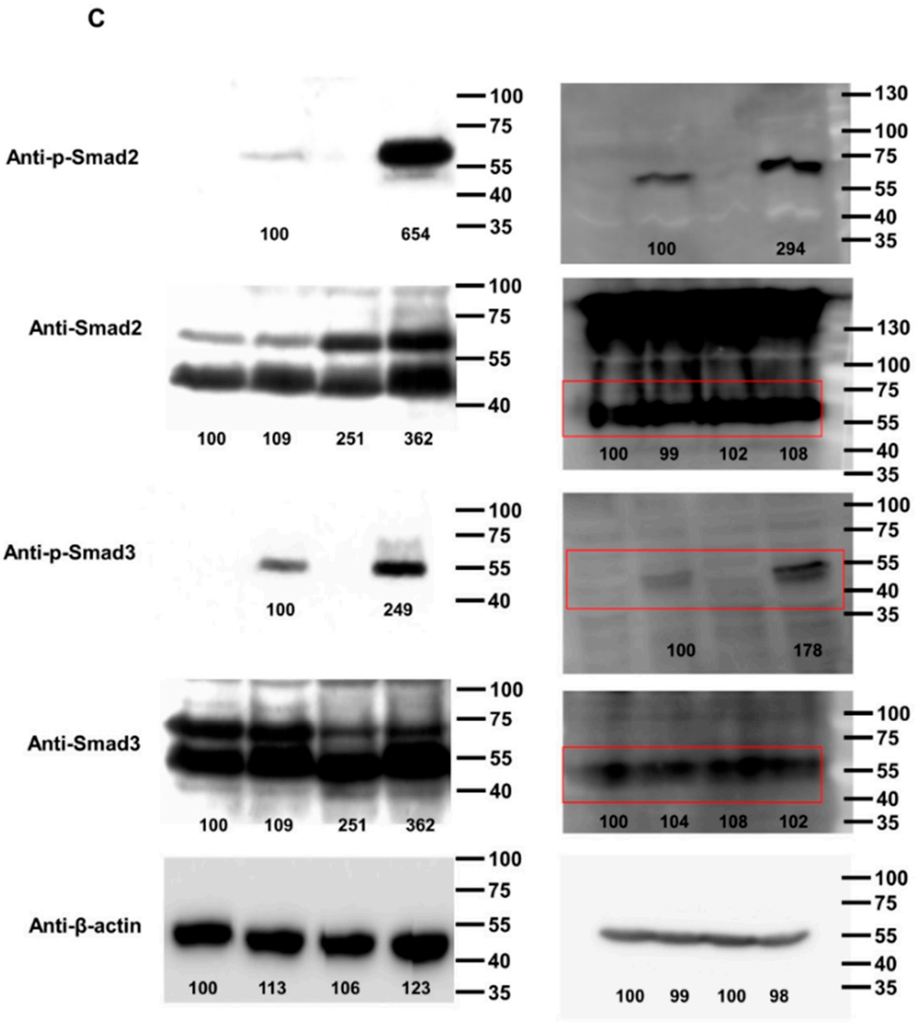

Detailed information about western blot of Figure 5C.

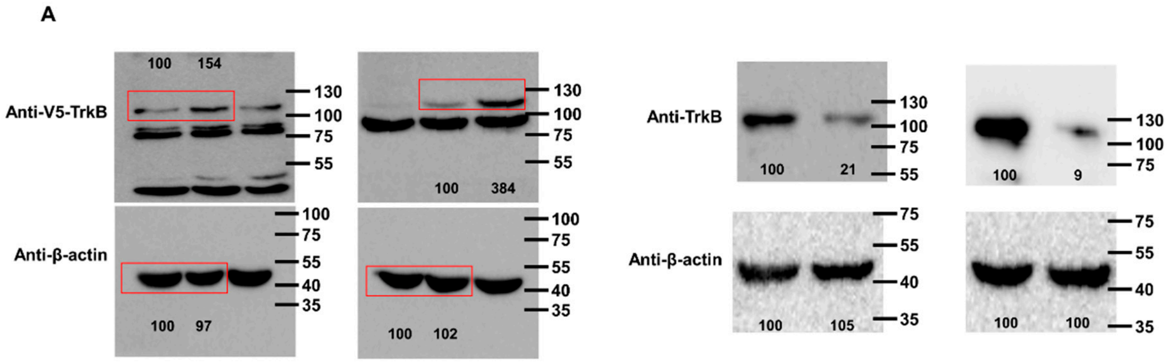

Detailed information about western blot of Figure S2A.

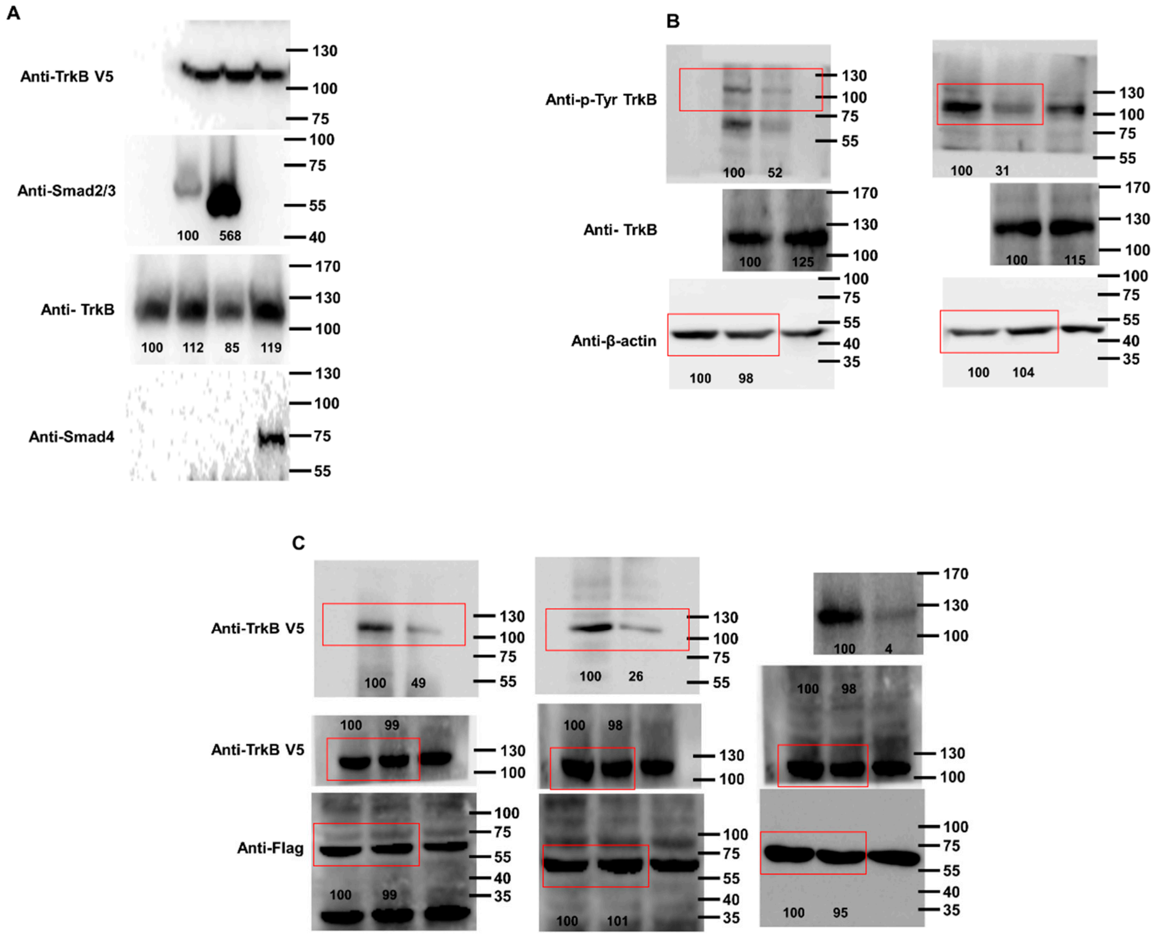

Detailed information about western blot of Figure S3A–C.

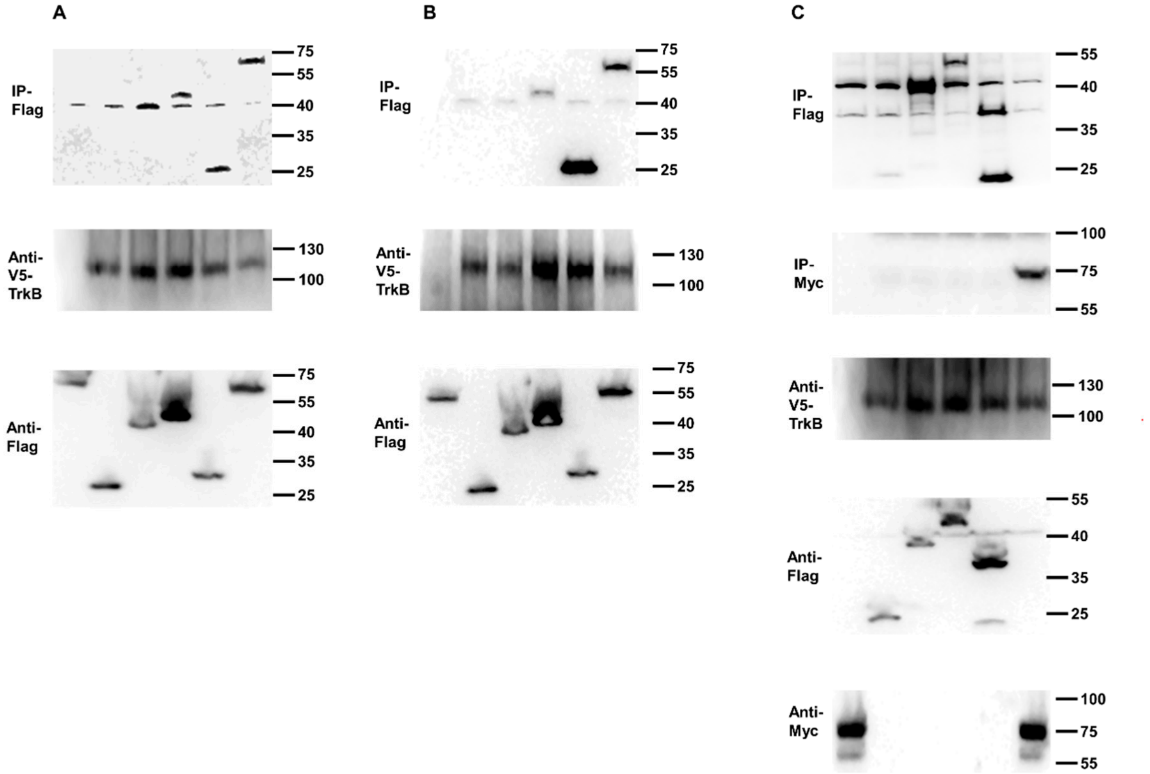

Detailed information about western blot of Figure S4A–C.

Table S1. Primer sequences for Cloning, RT-PCR.

Gene	Primer Sequence
	For site-directed mutagenesis
TrkB K588M	F: 5′-CTTGGTGGCAGTGATGACCCTGAAGGATGC-3′<br>R: 3′-GCATCCTTCAGGGTCATCACTGCCACCAAG-3′
	For RT-PCR
p15	F: 5′-CCAGAAGCAATCCAGGCGCG-3′<br>R: 5′-CGTTGGCAGCCTTCATCG-3′
p21	F: 5′-TGAGCCGCGACTGTGATG-3′<br>R: 5′-GTCTCGGTGACAAAGTCGAAGTT-3′
PAI-1	F: 5′-TGCATCGCCTGCCATTG-3′<br>R: 5′-GGACCTTGAGATAGGACAGTGCTT-3′
TMEPAI	F: 5′-CAGGAACTCAAAACCGTCAC-3′<br>R: 5′-AATTCACCCGGAGCAGTGAT-3′
β-actin	F: 5′-TCCCTGGAGAAGAGCTACGA-3′<br>R: 5′-AGCACTGTGTTGGCGTACAG-3′

© 2020 by the authors. Licensee MDPI, Basel, Switzerland. This article is an open access article distributed under the terms and conditions of the Creative Commons Attribution (CC BY) license (http://creativecommons.org/licenses/by/4.0/).
